# Empiric Treatment in HAP/VAP: “Don’t You Want to Take a Leap of Faith?”

**DOI:** 10.3390/antibiotics11030359

**Published:** 2022-03-08

**Authors:** Khalil Chaïbi, Gauthier Péan de Ponfilly, Laurent Dortet, Jean-Ralph Zahar, Benoît Pilmis

**Affiliations:** 1 Département de Réanimation Médico-Chirurgicale, AP-HP Hôpital Avicenne, Université Sorbonne Paris Nord, 93000 Bobigny, France; khalil.chaibi@aphp.fr; 2 Common and Rare Kidney Diseases, Sorbonne Université, INSERM, UMR-S 1155, 75020 Paris, France; 3 Service de Microbiologie Clinique, Groupe Hospitalier Paris Saint-Joseph, 75014 Paris, France; gpeandeponfilly@ghpsj.fr; 4 Institut Micalis, Unité Mixte de Recherche 1319, Université Paris-Saclay, INRAe, AgroParisTech, 92290 Châtenay Malabry, France; 5 Service de Bactériologie-Hygiène, CHU de Bicêtre, Assistance Publique des Hôpitaux de Paris, Centre National de Référence de la Résistance aux Antibiotiques, 94270 Le Kremlin-Bicêtre, France; laurent.dortet@aphp.fr; 6 INSERM UMR1184, Resist Unit, Université Paris-Saclay, 94270 Le Kremlin-Bicêtre, France; 7 Infection Control Unit, AP-HP Hôpital Avicenne, Université Sorbonne Paris Nord, 93000 Bobigny, France; jean-ralph.zahar@aphp.fr; 8 Équipe Mobile de Microbiologie Clinique, Groupe Hospitalier Paris Saint-Joseph, 75014 Paris, France

**Keywords:** antibiotic choices, HAP, VAP, colonization, antibiotic pressure

## Abstract

Ventilator-associated pneumonia is a frequent cause of ICU-acquired infections. These infections are associated with high morbidity and mortality. The increase in antibiotic resistance, particularly among Gram-negative bacilli, makes the choice of empiric antibiotic therapy complex for physicians. Multidrug-resistant organisms (MDROs) related infections are associated with a high risk of initial therapeutic inadequacy. It is, therefore, necessary to quickly identify the bacterial species involved and their susceptibility to antibiotics. New diagnostic tools have recently been commercialized to assist in the management of these infections. Moreover, the recent enrichment of the therapeutic arsenal effective on Gram-negative bacilli raises the question of their place in the therapeutic management of these infections. Most national and international guidelines recommend limiting their use to microbiologically documented infections. However, many clinical situations and, in particular, the knowledge of digestive or respiratory carriage by MDROs should lead to the discussion of the use of these new molecules, especially the new combinations with beta-lactamase inhibitors in empirical therapy. In this review, we present the current epidemiological data, particularly in terms of MDRO, as well as the clinical and microbiological elements that may be taken into account in the discussion of empirical antibiotic therapy for patients managed for ventilator-associated pneumonia.

## 1. Introduction

Ventilator-associated pneumonia (VAP) is one of the most frequent causes of intensive care unit (ICU)-acquired infections [1]. In French ICUs, 8% of patients developed hospital-acquired pneumonia in 2016, and 88.7% among them were ventilator-associated pneumonia [2]. 

For more than a decade, we have been confronted with the spread of multi-resistant bacteria in hospitals [3,4,5] and the community [6,7,8].

The increasing prevalence of resistance among bacteria, particularly gram-negative bacilli (GNB) and especially *Enterobacterales*, makes harder the choice of antibiotics, in case of infection. Several factors seem to be associated with a higher risk of infection related to multidrug-resistant organisms (MDRO), such as the local prevalence, previous antibiotic therapy, time of occurrence of the infection, and previous MDRO colonization.

In our clinical practice, the spread of MDRO resistance leads to a higher risk of antibiotic inadequation. Indeed, there are two opposing risks when choosing an empirical antibiotic therapy. On one side is the individual level, reflected by the risk to choose a narrow spectrum antibiotic with potentially important consequences in terms of mortality and morbidity. On the other side is the collective level, reflected by the choice of a broad-spectrum antibiotic which could contribute to the amplification of resistance.

In the last four years, medical research has been driven by the discovery of new beta-lactamase inhibitors and the marketing of new antibiotics with broad-spectrum activity.

Numerous authors suggest and encourage rational use of these new antibiotics out of fear of the emergence of new resistance mechanisms. However, certain clinical situations require empirical choices. It, therefore, seemed important to assess relevant factors and variables at the time of prescribing in order to choose the most appropriate molecule, both in terms of spectrum and possible ecological effects.

## 2. HCAP, HAP, VAP: Clinical Concepts and Historical Perspective

From the first consensus conference in 1996 until now, the history of concepts and definitions around nosocomial pneumonia has never been a long calm river. Its evolution is intrinsically linked to epidemiological, diagnostic, and therapeutic advances in the context of precision medicine [9]. A remarkable example would be the path of the Healthcare-Associated Pneumonia (HCAP) category in guidelines. If the term was initially defined in the 2005 ATS recommendations [10] in order to avoid inappropriate empiric antimicrobial therapy after the emergence of the “golden hours” concept in the intensive care unit (ICU) [11], it has finally proved to be irrelevant. By HCAP, it was meant “any patient who was hospitalized in an acute care hospital for 2 or more days within 90 days of the infection”. This categorization resulted in the increasing usage of broad-spectrum antibiotics in a population which eventually appeared to be no more infected with MDRO pathogens than patients with community-acquired pneumonia (CAP) [12]. Furthermore, this category failed to predict mortality [13]. The starting point was a retrospective cohort study based on more than 4000 patients with proven bacterial pneumonia within the 48 h following admission and transfer from a healthcare facility which showed an incidence of a quarter of admitted patients with Methicillin-Resistant *Staphylococcus aureus* (MRSA) infection, with the same incidence for *Pseudomonas aeruginosa* (PA) [14]. These findings, which led to treating CAP with risk factors of MDRO organisms carriage the same way as ventilator-associated pneumonia (VAP) regardless of the severity status, were later largely overruled [15]. Indeed, recent studies found no major difference in terms of bacterial epidemiology between CAP and HCAP [16], and antibiotic-resistant organisms were found to be rare whatever population at risk of carriage was analyzed [17].

Then, a deeper knowledge of bacterial epidemiology stood out as the key challenge of nosocomial pneumonia management for future years.

## 3. Definitions and Issues of Nosocomial Pneumonia

Hospital-Acquired Pneumonia (HAP) is defined as new pneumonia (a lower respiratory tract infection verified by the presence of a new pulmonary infiltrate on imaging) in non-intubated patients, that develops more than 48 h after admission. When it develops after 48 h of endotracheal intubation, it is categorized as a VAP. In the ICU, VAPs are the most present entity, with an overwhelming majority (more than 95%) of reported cases of pneumonia [18].

VAP is a major problem in the ICU due to its frequency and short-term consequences. It is the main source of healthcare-associated infections (HAI) in these departments, with an incidence reaching 40% of patients with up to 16 episodes per 1000 days of mechanical ventilation [19]. It is associated with significant morbidity since it is complicated in 30 to 50% of cases by septic shock, in 10 to 15% of cases by acute respiratory distress syndrome (ARDS), and in 10 to 15% of cases by multi-organ failure (MOF) [20,21,22,23,24]. It also has an impact on the duration of mechanical ventilation (increased from 7 to 11 days), the length of hospital stay (increased from 11 to 13 days), and health economics (EUR 30,000 to 40,000/per episode) [25,26,27]. Finally, the overall mortality of patients who experienced a VAP in the ICU is considerable (20 to 40% depending on the series), with an average attributable mortality (to the VAP episode alone) of 13% [28].

## 4. Epidemiology

HAP and VAP may be caused by a variety of pathogens and can be polymicrobial. Common pathogens include aerobic GNB (e.g., *Escherichia coli*, *Klebsiella pneumoniae*, *Enterobacter* spp., PA, *Acinetobacter* spp.) and Gram-positive cocci (e.g., *Staphylococcus aureus*, including MRSA, *Streptococcus* spp.) [29,30]. Furthermore, there is increasing recognition that a substantial fraction of nosocomial pneumonia may be due to viruses [29].

Among 8474 cases of VAP reported in the United States Centers for Disease Control and Prevention, the distribution of pathogens associated were *Staphylococcus aureus*, *Pseudomonas aeruginosa*, *Klebsiella* species, *Enterobacter* species, *Acinetobacter baumannii*, and *E. coli* in, respectively, 24.1%, 16.6%, 10.1%, 8.6%, 6.6%, and 5.9% of cases [31]. In a prospective observational study evaluating 158,519 patients admitted to the University of North Carolina Hospital over a 4-year period, a total of 282 episodes of documented VAP and 190 episodes of documented HAP in non-ventilated patients were identified (Table 1) [32].

These findings are similar to those observed in a meta-analysis of 24 studies performed during the development of the 2016 Infectious Disease Society of America guidelines [33].

The etiology of HAP and VAP depends largely upon whether the patient has risk factors for MDRO pathogens [33]. The frequency of specific MDRO pathogens varies among hospitals, within hospitals, and between different patient populations. One of the major problems lies in the spread of MDROs and, specifically, extended-spectrum beta-lactamase-producing *Enterobacterales* (ESBLEs) in the community. This diffusion has consequences in hospitals and in intensive care units where, among hospitalized patients, between 5 and 25% are ESBL producing *Enterobacterales* carriers [34,35,36].

### 4.1. Bacterial Epidemiology: A Practical Approach in ICU

**Figure 1:**
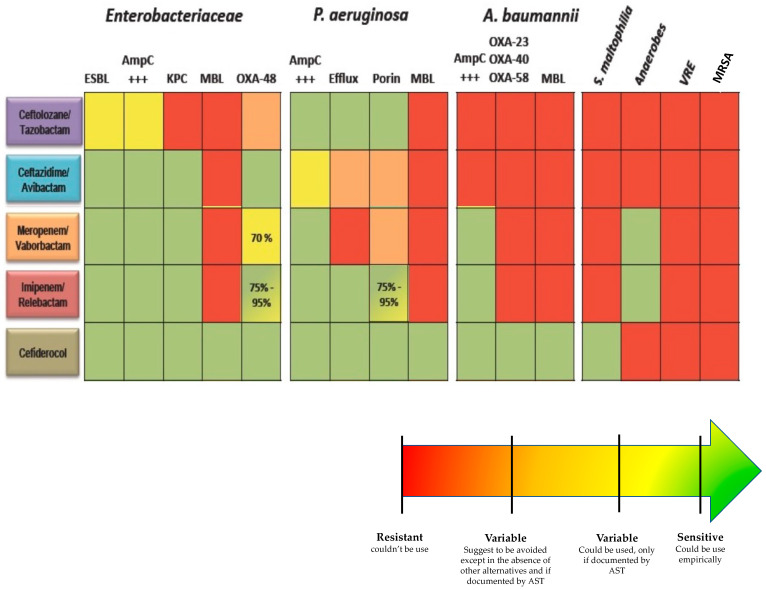
Spectrum of activity of new antibiotics. AST: Antibiotic susceptibility test, ESBL: Extended-spectrum beta-lactamase, AmpC: Cephalosporinase, KPC: *Klebsiella pneumoniae* carbapenemase, MBL: metallo-betalactamase, VRE: vancomycin resistant Enterococci, MRSA: Methicillin-resistant *Staphylococcus aureus*.

Choosing the right antibiotic lies in anticipating both the species and the resistance mechanisms that will be involved in the infection (Figure 1). If the local epidemiology weighs heavily on the species involved, it remains that certain clinical situations and data specific to the medical history of the patient must lead to considering certain species.

### 4.2. Profiling Bacterial Species in Pneumonia: Born to Be Wild

#### 4.2.1. Methicillin Susceptible *Staphylococcus aureus* (MSSA)

Among the population, 30 to 50% are permanently or intermittently colonized with *Staphylococcus aureus* species. Moreover, the risk of secondary infection seems to be higher among previously colonized patients [37,38,39]. Indeed, in 2017 post-hoc analysis of two cohort studies of more than 9000 critically ill patients found that patients colonized with *Staphylococcus aureus* at ICU admission had an up to 15 times increased risk for developing this outcome compared with non-colonized patients [40]. *Staphylococcus aureus* should be considered in the case of HAP that complicates influenza infection or in the case of early HAP in patients known to be previously colonized with MSSA. Several authors suggested a higher risk in a specific population, such as traumatic and non-traumatic brain injury patients [41]. Indeed, the authors suggested that MSSA is most frequent in this specific population, accounting for up to 40–50% of VAP. In a recent study focusing specifically on bacteriological aspects of *Staphylococcus aureus* VAP, the authors highlighted that nearly 74% of the patients had severe head trauma or a *priori* history of coma [41,42].

#### 4.2.2. *Enterobacterales*

*Enterobacterales* remain the most frequent species found in HAP and VAP patients. These species must be systematically considered in the choice of antibiotics, whatever the circumstances. The only question that should be asked is if the antibiotic spectrum should include resistant bacteria. In this perspective, the time of occurrence of the event should be addressed as major information. Indeed, early HAP and VAP seem to be related to sensitive species, whereas the duration of hospitalization and previous antibiotic therapy seem to be associated with more resistant species [43].

#### 4.2.3. *Pseudomonas aeruginosa*

Assessing the specific risk of PA infection is highly relevant since the mortality attributable to this GNB seems more important than others (both because of the multi-resistant nature of the germ inducing a delay in appropriate antibiotic therapy and because it more often affects more severe patients) [44]. *Pseudomonas aeruginosa* colonization remains rare even in critically ill patients in the ICU. Indeed, in developed countries, the PA colonization rate at ICU admission is close to 10% in several studies [45,46,47]. However, PA is a highly prevalent causative pathogen in HAP and VAP. An experience of the French national surveillance, REA-RAISIN, found that a higher probability of PA-VAP is regularly associated with higher age, length of mechanical ventilation before pneumonia, antibiotics at admission, and admission in a ward with a higher incidence of patients with PA infection. Interestingly, transfer from a medical unit or ICU was also found to be associated with a higher probability of PA-VAP (45). Hospital admission should thus be considered a turning point in the colonization pressure experienced by the patient (and not only the admission in ICU). Lower probability of PA pneumonia was associated with traumatism and, as expected, with admission in a ward with higher patient turnover. Some populations seem to be more at risk, such as patients with COPD, cystic fibrosis, or bronchiectasis. Old studies suggested a higher prevalence of late VAP [48]. Indeed, as PA is a saprophytic species specifically linked to water, acquisition requires a contaminated environment and selection pressure.

#### 4.2.4. *Acinetobacter baumannii*

Although found with a low worldwide prevalence, *Acinetobacter baumannii* is one of the most antibiotic-resistant pathogens, with 50% of carbapenem-resistant isolates in US intensive care units, including a vast majority of extreme drug-resistant (XDR) strains [49]. Moreover, the survival of Acinetobacter baumanii in the biofilm makes their treatment difficult [50,51].

*Acinetobacter* are ubiquitous organisms recovered from soil or surface water. *Acinetobacter* are rarely found in the microbiota of patients in the northern hemisphere. Indeed, several studies suggested a low rate of *Acinetobacter baumannii* carriage in the communities in Germany and France [52], even in the population of patients admitted to the intensive care unit [53]. However, authors highlighted higher carriage rates in other parts of the globe, such as Hong Kong, the Asia-Pacific region, and other countries with hot and humid climates. In these locations, *Acinetobacter* *baumannii* has emerged as a cause of severe community-acquired infections [54]. Classically-found risk factors for *Acinetobacter baumannii* infection are tropical or sub-tropical climate, excessive alcohol consumption, smoking, or having an underlying health condition (diabetes mellitus or chronic lung disease) with a different weight of each risk depending on the location [55]. In the ICU, a difference must be made according to the epidemiological data. Whereas *Acinetobacter baumannii* is associated with early-onset HAP/VAP in southern countries, it seems rarely isolated in northern countries and is usually associated with several risk factors. Indeed, factors independently associated with *Acinetobacter baumannii* infection are commonly found to be immunosuppression, previous antimicrobial therapy, previous sepsis in the ICU, and a history of recent invasive procedures [56].

#### 4.2.5. *Stenotrophomonas maltophilia*

HAP or VAP related to *Stenotrophomonas maltophilia* (SM) are rare [30]. SM is an environmental bacterium found in aqueous habitats, including plant rhizospheres, animals, foods, and water sources. It is not a highly virulent pathogen, but it has emerged as an important nosocomial pathogen. The incidence of SM hospital-acquired infections (HAI) is increasing, particularly in the immunocompromised patient population [57,58]. Risk factors for this infection include chronic respiratory diseases (especially cystic fibrosis), hematologic malignancy, chemotherapy-induced neutropenia, organ transplant patients, human immunodeficiency virus (HIV) infection, hemodialysis patients, and neonates [59]. Furthermore, hospital settings, prolonged intensive care unit stays, mechanical ventilation, tracheostomies, central venous catheters, severe traumatic injuries, significant burns, mucositis or mucosal barrier damaging factors, and the use of broad-spectrum antibiotic courses were shown to increase the risk of infection [60,61].

### 4.3. Risk Factors Associated with MDRO

**Figure 2:**
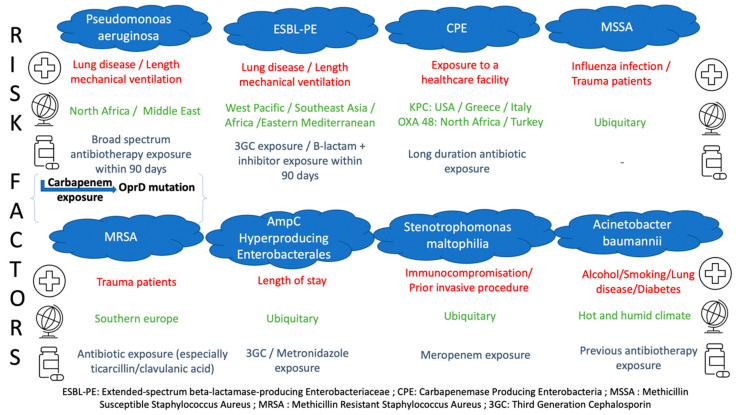
Risk factors for MDRO-related infections.

As highlighted before, three main factors seem to be associated with MDRO-related pulmonary infection. Firstly, previous antibiotic therapy is one of the major risk factors as it is the source of selecting/inducing MDRO, and it paves the field of acquisition of resistant bacteria from the environment. Thus, before considering a species or a particular resistance mechanism, it is essential to trace the history of specific antibiotic exposure that can help the practitioner assess the risk of dealing with a specific species or resistance mechanism (Figure 2). For instance, carbapenem exposure and exposure to β-lactams inactive against *Pseudomonas aeruginosa* have been strongly correlated to the emergence of Carbapenem-Resistant *Pseudomonas aeruginosa* isolation, due to the repression or inactivation of the OprD gene encoding porin OprD2 [62,63]. As for species, *Stenotrophomas maltophilia*-related pneumonia has been found to be associated with previous exposure to Meropenem [64,65] and *Enterococcus* with previous exposure to third-generation cephalosporins [66].

Secondly, the length of hospitalization seems to be an important risk factor as it corresponds to a duration of exposure to a particular (bacterial) environment, responsible for the modification of the patients’ microbiota [67]. Indeed, several authors suggested [68,69] that duration of hospitalization and antibiotic therapy were the two main factors associated with MDRO-related pneumonia.

Thirdly, prior colonization with MDROs seems to be an indispensable prerequisite for the occurrence of MDRO infection [37,70,71]. In light of the spread of MDRO and the increase in the number of colonized patients [35], it seems more and more difficult to interpret the weight of MDRO colonization as a risk factor for MDRO infection. Several authors suggested that rare are the patients infected with MDRO among carriers [72,73]. In a prospective study among carriers, only 6% developed ICU-acquired pneumonia related to ESBL producing *Enterobacterales.*

A large study conducted in a French ICU suggested that the first infection episode rates in EBSL-PE carriers vary from 10% to 42% [70,74,75,76]. Whereas, the rate of the second episode rises from 10% to 30% [73]. *Klebsiella pneumoniae* carriage has also been found to carry a specific risk of colonization/infection transition in a surgical population of liver transplanted patients [77]. As for antibiotic use, a carbapenem exposure within the preceding three days has been reported to have a protective effect on ESBL PE VAP in one study [78]. Finally, a retrospective cohort study of more than 500 ICU patients with suspected VAP analyzed sensitivity and specificity of prior ESBL-PE colonization as a predictor of ESBL-PE-VAP and found, respectively, 85.0% and 95.7%. The positive and negative predictive values were 41.5% and 99.4%, respectively, with a positive likelihood ratio of 19.8. Moreover, no data support an impact of ESBL carriage on mortality which is a supplementary argument in favor of a “wait and see” strategy [79].

It, therefore, seems necessary to be able to identify among patients carrying multidrug-resistant bacteria those at risk of infection. Studies that occurred outside the ICU [80] and in the ICU [81,82] have suggested that relative abundance was the main risk factor associated with secondary bacteremia and VAP. Besides the risk due to the significant biomass of multidrug-resistant bacteria, it seems that colonization with non-*E. coli* species was associated with a higher risk of secondary infection [82]. Indeed, it has been shown that a high relative fecal abundance of ESBL-producing *Enterobacterales* is associated with a higher risk of ESBL-producing *Enterobacterales* associated VAP [81]. One study showed that in ICU patients colonized with ESBL-producing *Enterobacterales*, the onset of ESBL-producing *Enterobacterales* throat carriage preceded the occurrence of ESBL-producing *Enterobacterales* associated VAP [82].

### 4.4. Extended-Spectrum Beta-Lactamase-Producing Enterobacterales (ESBL-PE)

A recent meta-analysis found an overall prevalence of ESBL-producing *Enterobacterales* in a community of 14% among healthy individuals with an increasing annual rate of approximately 5%. The most impacted locations were in the West Pacific, Southeast Asia, Africa, and the eastern Mediterranean [83]. In Europe, Italy has a particularly high rate of ESBL, with 26% of *Escherichia coli* displaying resistance to the third-generation cephalosporins in 2013 [84]. Within a country, the prevalence can be very different from one region to another; in some locations, we observed the endemic situation in the community [35], whereas others were scarcely affected [35]. A 2012 French prospective study in a medical ICU showed a 15% ESBL-producing *Enterobacterales* carriage rate, mostly of *Escherichia coli* (62%). Transfer from another ICU, previous hospital admission in another country, surgery within the past year, prior neurologic disease, and prior administration of third-generation cephalosporin (within 3–12 months before ICU admission) have been found to be risk factors of ESBL-producing *Enterobacterales* carriage at ICU admission. Furthermore, advanced age, male gender, colonization pressure (defined as the sum of the daily proportion of patients in the unit colonized with ESBL-PE during the days preceding acquisition or ICU discharge), 3GC within the past three months, and B-lactam + inhibitor within three months were associated with the ESBL-PE-acquired carriage in the ICU in the same study [73].

### 4.5. AmpC Hyperproducing Enterobacterales (AHE)

In a retrospective study of more than a thousand ICU patients, the prevalence of intestinal colonization with AHE evolved from 2% at admission to 30% in patients with lengths of stay (LOS) exceeding four weeks. Metronidazole, cephalosporin use, and the LOS were found to be independently associated with acquired carriage in ICU patients [85]. It has been known for over 50 years that commensal anaerobes confer protection against exogenous pathogens, which may explain why metronidazole, by its impact on colonization resistance, favors the emergence of such mutants from subdominant, wild-type *Enterobacterales* populations. Therefore, AHE community prevalence could be considered insignificant, whereas its emergence in the ICU should not. Currently, information on the digestive carriage of AHE is not systematically provided to the clinician and differs from one center to another, even though studies have shown the value of this information for initial therapeutic adequacy in the case of sepsis [86].

### 4.6. Carbapenemase-Producing Enterobacterales (CPE)

The distribution of the different types of CPE is very heterogeneous on a global scale [87]. In communities, *Klebsiella-producing Carbapenemase* (KPC) is widespread in the United States and endemic in some European countries, such as Greece and Italy [88]. Among the metallo-β-lactamases (MBL), New Delhi metallo-β-lactamase (NDM), Verona integron-encoded metallo-β-lactamase (VIM), and imipenemase metallo-β-lactamase (IMP) enzymes are the most frequently identified worldwide [89]. IMP producing Gram-negative bacteria are mainly located in eastern Asia and Australia, mostly in *Acinetobacter baumannii*. VIM producers are most often found in Italy and Greece (*Enterobacterales*) and in Russia (*Pseudomonas aeruginosa*) [90,91]. OXA-48–producing *Enterobacterales* are endemic in Turkey and are frequently encountered in several European countries and across North Africa [92]. Reported risk factors for community carriage of CPE are, as expected, geographical location and recent antibiotic use. In the ICU, the prevalence of CPE varies from 6% to 37%, depending on the unit location [93,94]. A recent five-year case control study found the length of hospital admission >20 days, hospital admission within the previous year, exposure to a healthcare facility in a country with high carbapenem-resistant *Enterobacterales* prevalence 3 months before admission, and the use of antibiotics longer than 10 days to be independent predictors of CPE carriage.

### 4.7. Methicillin-Resistant Staphylococcus aureus (MRSA)

Several risk factors of MRSA acquisition during a hospital stay have been described as LOS, presence of patients colonized with MRSA in the same ICU at the same time, previous antibiotic use (especially ticarcillin/clavulanic acid), central venous catheter insertion, and period of nurse understaffing [95,96]. Interestingly, the specific population of trauma patients have been found to be particularly at risk of MRSA acquisition. In this very population, road traffic accident victims were at greater risk of acquiring MRSA than patients who had suffered other mechanisms of injury, probably because of more skin defects, such as open versus closed fractures, or more surgical procedures [97].

## 5. When to Use Broad-Spectrum Antibiotics, What Tools to Guide Us?

Colonization of the upper respiratory tract is a precondition to VAP in almost all patients [98]. However, prior colonization is not systematically responsible for the infection. Carriage should be interpreted solely as a risk factor as it could not be responsible for an infection on its own [99,100]. It is important to emphasize this point, considering the fact that knowledge of colonization misleads physicians in an overprescribing path [101].

### 5.1. Moving from an Empirical to Oriented Antimicrobial Choices

The conventional microbiological approach for HAP-VAP diagnosis consisted of cultures coupled with antimicrobial susceptibility testing, requiring approximately 48 h to 72 h from sampling to results delivery to physicians. It is important to notice that the implementation of MALDI-TOF MS in microbiology laboratories has already shown an impact on HAP-VAP management [102].

New strategies need to be implemented to reduce the pathogen identification time and MDRO-genes because of frequently unappropriated empirical therapy. Molecular techniques, such as syndromic m-PCR panels, have introduced a considerable change in antibiomicrobial stewardship intervention, accelerating targeted therapy in different conditions such as HAP-VAP.

Among these, the BioFire^®^ FilmArray^®^ Pneumonia Panel (FA-PN) (bioMérieux SA, Marcy-l’Étoile, France) is the widely used one. It is a Food and Drug Administration (FDA) syndromic m-PCR that simultaneously identifies 33 targets: 15 typical and 3 atypical bacterial pathogens, 8 respiratory viruses and 7 resistance genes in BAL/mini-BAL, tracheal aspirates (ETA), and sputum specimens.

Two recent multicentric studies on performance evaluation demonstrated excellent positive percentage agreement and negative percentage agreement values when compared with conventional culture methods [103,104]. In all these studies, it is important that the prevalence of bacteria off-panel is non-negligible and should be kept in mind by physicians and laboratory staff.

Many prospective and retrospective studies, so-called “real-life studies”, have been published to evaluate the clinical impact of this approach. Caméléna et al. showed a considerably reduced sample-to-result time compared to conventional approach (5.5 h vs. 25.9 h for cultures (*p* < 0.001) and 57 h for AST (*p* < 0.001), respectively) [105]. During the COVID-19 pandemic, Maataaoui et al. revealed in a prospective cohort of 112 episodes (104 HAP-VAP) an early empirical therapeutic change in 34% of HAP-VAP episodes (of which 46.3% were withdrawn) when this panel was performed [106]. Another recent prospective study showed among COVID-19 ICU patients that antibiotics were initiated in 87 (72.5%) of 120 pneumonia episodes and were not administered in 80 (87.0%) of 92 non-pneumonia episodes based on FA results [107].

Because of the high cost of this approach, some studies suggested scores to rationalize performing such a test. A comprehensive study suggested that both clinical (temperature and Clinical Pulmonary Infection Score) and biological parameters (WBC BAL count and % of PMNs) were correlated with FA-PN with or without conventional culture results [108]. In some cases, interpretation of results remains a challenge for physicians and laboratory staff. Based on a retrospective non-interventional study, Novy et al. designed an algorithm helping antimicrobial stewardship prescription with FA-PN results in cases of HAP-VAP suspicion and confirmed the poor reliability of ETA samples because of over-detection of the microbial and viral genome [109].

Other panels exist, such as the syndromic m-PCR panel for HAP-VAP developed by Curetis (Curetis GmbH, Holzgerlingen, Germany) with The Unyvero P55 Pneumonia panel, capable of identifying 20 pathogens of lower respiratory tract infections (LRTI) and 19 resistance genes. With a longer turn-around time of 5 h, performances seemed to be lower than previously described panels. However, a non-interventional study recently showed that this test could have led to modifications of empirical therapy in 60% (57/95) of HAP-VAP episodes [110]. “In-house” multiplex PCRs have been customized by several laboratories, such as the custom-designed multi-pathogen TaqMan Array Cards (TAC; Thermo Fisher Scientific, Waltham, MA, USA) in a UK center with good analytical performance [111].

This kind of approach has the advantage of identifying multiple pathogens with a shorter turn-around time, including those which are fastidious and pathogens that cannot be retrieved by conventional cultures. This remains particularly true when antimicrobials have already been started before sampling.

However, the reliance on the presence of resistance genes should be interpreted with caution. Importantly, these methods can only detect antibiotic resistance genes which have been chosen by industrials, and the limits of these assays need to be well known by physicians and microbiology labs. Conventional culture methods shall be continued because of the significant prevalence of uncovered pathogens.

### 5.2. How to Choose the Empirical Antibiotic: “Because It Was Him, Because It Was Me”

The ICU carries multiple specificities making the choice of empiric antimicrobial therapy a singular decision for each patient. Indeed, multiple parameters must be considered for critically ill patients, including the severity of illness, the seriousness of the situation, the certainty of the diagnosis, the local microbial ecology, and MDR prevalence in the unit. However, once these parameters are settled, the first legitimate question would be: does the treatment have to be empirical?

### 5.3. Rusher or Dragger?

Delayed initiation of antibiotic therapy has often been cited as a major risk factor for excess mortality, supporting the idea that “a large antimicrobial spectrum” should be provided to ICU patients. As the global rise of MDR incidence has become more widely known among practitioners, a “structural” tension has arisen between the need not to delay antibiotic therapy and the need to choose the right one. The idea that all ICU patients should be started on antibiotics as soon as possible implies that all patients admitted in those units have the same level of severity which is obviously inaccurate [112]. Studies have shown that this increased risk of mortality due to delayed initiation of antibiotic therapy was effective, especially for the most severe patients [113]. It would, therefore, seem appropriate, when the patient’s condition allows it, to wait for the germ identification and antibiogram.

### 5.4. Under Pressure 

Local epidemiological knowledge is crucial. As seen previously, there is a heterogeneity in the distribution of MDRs, which suggests different considerations when choosing antibiotics, depending on the unit location [114]. If MDR carriage does not mandate any antibiotic therapy, it is well documented that it is a necessary step prior to infection [115]. Hence, each practitioner should be aware of the bacterial epidemiology of the hospital and unit in which they work [33].

Colonization pressure described in 1994 by Bonten et al. [116] is a fundamental concept that needs to be addressed in order to choose an adequate antibiotic. A study led by Trouillet et al. in 1998 was the first trial to link the changing bacterial epidemiology according to mechanical ventilation (MV) duration and previous antibiotic therapy. Firstly, it showed that patients who were not exposed to antibiotics and who underwent MV for less than seven days (in other terms, patients who had very low colonization pressure) were infected with the “usual” germs present in oropharyngeal and respiratory microbiota (*Streptococcus*, *Haemophilus influenzae*, *Enterobacterales*). On the other hand, when they underwent MV for more than seven days and had greater antibiotic exposure, non-fermenting gram-negative bacilli (PA, *Stenotrophomonas*, *Acinetobacter*) were more frequent. Multiple lessons could be drawn from this study with a remarkable reproducibility in the following years. It showed the importance of colonization pressure in VAP bacterial epidemiology and raised awareness about the fundamtental importance of selection pressure, which has been later confirmed in other studies [117]. MDR prevalence and antibiotic usage have risen in the 20 years since this historical study. If the “five days after ICU admission cut off” is since then usually used to materialize the risk of resistance [111,118], shifting the clinical and epidemiological reasoning in time could be an appropriate current adaptation. Indeed, according to geographical locations and hospital epidemiological situations, the patient admitted to the ICU or already in the ICU and undergoing MV could have experienced colonization and selection pressure for several days before. Thus, considering this pressure from the first contact with the healthcare facility (Emergency Room, medical ward, ICU), the question “what antibiotic should I use for this VAP?” as a continuum might be more relevant.

**Figure 3:**
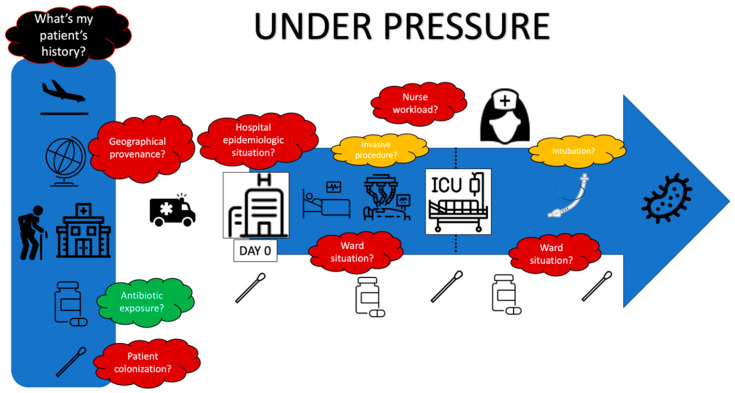
Under pressure.

In conclusion, the reasoned choice of antibiotics to treat HAP/VAP requires the consideration of many variables ranging from local epidemiological data to the patient’s personal history, including prior antibiotic therapy and length of stay (Figure 3). The new microbiological diagnostic methods make it possible to move from an empirical prescription to an oriented prescription, reducing the delay for adequate antibiotic therapy.

## Figures and Tables

**Table 1:** Frequency of isolation of pathogens from patients with Ventilator-Associated Pneumonia (VAP) and non-ventilated patients with Hospital-Acquired Pneumonia (HAP) [32].

	Ventilator-Associated Pneumonia	Healthcare-Associated Pneumonia
**Gram-positive cocci**	**39.3%**	**55.8%**
*Staphylococcus aureus* (SA)	36.8%	47.9%
Methicillin Resistant SA	24.4%	28.9%
Methicillin Susceptible SA	12.4%	19%
*Streptococcus pneumoniae*	2.5%	7.9%
**Gram-negative bacilli**	**60.7%**	**44.2%**
*Enterobacter* sp.	3.2%	4.3%
*Escherichia coli*	3.5%	4.3%
*Klebsiella pneumoniae*	2.1%	6.8%
*Serratia marcescens*	2.8%	2.6%
*Pseudomonas aeruginosa*	21.3%	13.1%
*Stenotrophomonas maltophilia*	8.8%	1.6%
*Acinetobacter* spp.	10.4%	4.7%
Other species	8.6%	6.8%
